# Protocol for untargeted LC-MS/MS metabolomics annotation and differential abundance analysis using the SIRIUS Python client

**DOI:** 10.1016/j.xpro.2026.104771

**Published:** 2026-08-07

**Authors:** Jonas A. Emmert, Sebastian Böcker, Markus Fleischauer

**Affiliations:** 1 Chair for Bioinformatics, Institute for Computer Science, Friedrich Schiller University Jena, Hans-Knöll-Straße 6, 07745 Jena, Thuringia, Germany; 2 International Max Planck Research School “Chemical Communication in Ecological Systems”, Max Planck Institute for Chemical Ecology, Hans-Knöll-Straße 6, 07745 Jena, Thuringia, Germany; 3 Bright Giant GmbH, Hans-Knöll-Straße 6, 07745 Jena, Thuringia, Germany

**Keywords:** Bioinformatics, Metabolomics, Special Issue, Protocols in Metabolomics and Lipidomics

## Abstract

We present a protocol for programmatic untargeted LC-MS/MS metabolomics using PySirius, the Python client for SIRIUS. We describe steps for importing data with LC-MS feature alignment, and annotating features with molecular formulas, structures, and compound classes. We then detail procedures for analyzing differential abundance by fold change between groups and visualizing results. As proof of concept, we replicate the finding that rosmarinic acid is more abundant in old than young rosemary (*Rosmarinus officinalis*) leaves.

## Highlights

• Steps to import LC-MS/MS data with blank subtraction and feature quality filtering
• Steps for automated molecular formula, structure, and compound class annotation
• Guide to tagging samples and computing feature- and compound class-level fold changes
• Full Python workflow with visualization allowing integration with downstream libraries

## Abstract (editor highlights)

Publisher’s note: Undertaking any experimental protocol requires adherence to local institutional guidelines for laboratory safety and ethics.

## Graphical abstract

**Figure:**
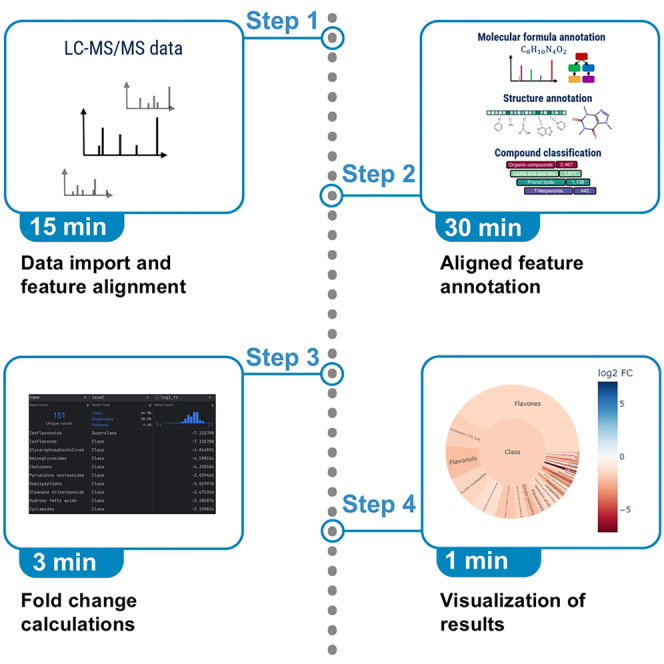

## Before you begin

Metabolomics, the comprehensive analysis of metabolites in a biological organism or system, provides a functional readout of its physiological state. Untargeted metabolomics by liquid chromatography (LC) coupled tandem mass spectrometry (MS/MS or MS2) uses collision-induced dissociation to generate complex datasets. In many cases, the vast majority of detected features lack structural annotation, due to the inevitable incompleteness of spectral libraries. The SIRIUS platform addresses this challenge by integrating molecular formula annotation from isotope patterns and fragmentation trees,^1^^,^^2^^,^^3^ structure annotation via CSI:FingerID,^4^ and compound class annotation via CANOPUS^5^ into a single computational framework. SIRIUS was initially developed to determine the molecular formula of a query compound without the need for any database lookup^6^^,^^7^ but later became a framework and graphical user interface to access computational methods as well as results.^8^ PySirius allows users to access the full SIRIUS functionality through a RESTful (REpresentational State Transfer) Application Programming Interface (API). This enables end-to-end metabolomics workflows from raw data import to annotated differential abundance tables, all within a single Python environment such as a Jupyter notebook.

The protocol below describes the specific computational steps used to analyze an LC-MS/MS dataset of rosemary (*Rosmarinus officinalis*) tissue sampled across different plant parts and developmental zones.^9^ As a concrete validation task, we replicate the finding that rosmarinic acid is more abundant in old than in young leaf tissue.^9^^,^^10^^,^^11^

This protocol is not meant to provide any type of evaluation of the underlying computational methods. For validations, statistics, and discussions of the methods integrated in SIRIUS, we refer to the above-mentioned publications. Whereas this protocol focuses on fold change analysis and comparing abundances between groups, it must be understood that a full analysis should also consider statistical significance testing, uncertainty estimation, replicate variability, and multiple-testing considerations. Fold changes alone can be misleading if not paired with measures of dispersion and confidence.

### SIRIUS account registration

SIRIUS is free for academic use. A one-time registration at https://portal.bright-giant.com/auth/register/is required to obtain login credentials.

### Dataset download

The example dataset consists of mzXML files from a metabolomics study of rosemary tissue, publicly available on MassIVE: MSV000080553. Files are organized by plant part (leaf, stem, flower) and developmental zone (Z1–Z10). A flat key=value metadata file mapping group names to filenames is provided within the dataset. This protocol assumes that the directory of the downloaded data is named after its accession number, and is placed in the same parent folder as the code using it.

The dataset can be downloaded from the MassIVE FTP server using Python’s built-in ftplib. The function below recursively mirrors the remote directory, preserving the folder structure locally.

import ftplib

import os

def download_ftp_dir(ftp, remote_dir, local_dir):

 os.makedirs(local_dir, exist_ok=True)

 lines = []

 ftp.retrlines(f”LIST {remote_dir}”, lines.append)

 for line in lines:

  parts = line.split(maxsplit=8)

  kind = line[0]

  name = parts[8].split(“ -> )[0]

  remote_path = f”{remote_dir}/{name}”

  local_path = os.path.join(local_dir, name)

  if kind in (“d”, “l”):

   download_ftp_dir(ftp, remote_path, local_path)

  else:

   with open(local_path, “wb”) as f:

    ftp.retrbinary(f”RETR {remote_path}”, f.write)

ftp = ftplib.FTP(“massive-ftp.ucsd.edu”)

ftp.login()

download_ftp_dir(ftp, “/v01/MSV000080553”, “./MSV000080553”)

ftp.quit()

### Innovation

Computational metabolomics workflows commonly involve multiple tools that exchange data through intermediate files, making end-to-end analyses difficult to reproduce and share. The SIRIUS REST API, introduced with SIRIUS 6, decouples the storage layer from the access layer and exposes the full analytical capability of SIRIUS through a standardized, OpenAPI-compliant programmatic interface. PySirius, the Python client library built on this API, brings these capabilities into standard Python data analysis environments for the first time. This protocol demonstrates how PySirius enables a complete and self-contained metabolomics analysis notebook: from raw LC-MS/MS data import, through quality-aware feature filtering and SIRIUS annotation, to feature- and class-level fold change computation and visualization. Because all steps are expressed as Python function calls over the REST API rather than as file manipulations or command-line invocations, the workflow is inherently portable. The same notebook can be executed on any machine with PySirius installed, and the analysis configuration can be saved directly into the SIRIUS project space, making it auditable and reproducible.

## Key resources table

**Table:** 

REAGENT or RESOURCE	SOURCE	IDENTIFIER
**Deposited data**		

Rosmarinus officinalis	MassIVE	MSV000080553

**Software and algorithms**		

Python	Python.org	RRID:SCR_008394
pandas	PyPI	RRID:SCR_018214
plotly	PyPI	RRID:SCR_013991
Conda	Anaconda, Inc.	RRID:SCR_018317
pubchempy^12^	PyPI	https://github.com/mcs07/PubChemPy DOI: https://doi.org/10.5281/zenodo.16754840
PySirius	GitHub	https://github.com/sirius-ms/sirius-client-openAPI DOI: https://doi.org/10.5281/zenodo.21257821
SIRIUS	GitHub	https://github.com/sirius-ms/sirius DOI: https://doi.org/10.5281/zenodo.21257821

## Materials and equipment

### Install software dependencies

Install conda on your system and download SIRIUS. Create a conda environment with Python, activate it, and install all other dependencies.

conda create -n star_rosemary python

conda activate star_rosemary

pip install “git+https://github.com/sirius-ms/sirius-client-openAPI@star-protocol#subdirectory=client-api_python/generated”

pip install pandas plotly pubchempy

### System requirements and used system

SIRIUS supports Linux, Windows, and macOS operating systems. A stable internet connection is central to being able to use all SIRIUS functionality. The protocol was tested on both Windows and Linux.

The times reported in this protocol are achieved using a Dell XPS 15 9530 laptop running Windows 11 with 32GB DDR5 RAM and an Intel Core i7 13700H CPU. The CPU contains eight efficiency cores, each with one thread, and six performance cores supporting two threads each, totaling 14 cores supporting 20 threads.

### Exact software dependency versions

Should problems arise with the software dependencies, please try these exact versions. We additionally provide conda environment files; see data and code availability.

**Table:** 

Package	Installation source	Version
Python	conda	3.14.4
pandas	PyPI	3.0.2
plotly	PyPI	6.7.0
pubchempy^12^	PyPI	1.0.5
PySirius	GitHub	6.5.0
SIRIUS	GitHub	6.5.0

### Dataset specifications and impact on SIRIUS

The rosemary dataset contains 47 mzXML files, each about 30 MB in size. The complete dataset of 1.42 GB contains 41126 aligned features, of which 6307 contain MS/MS data. Only features with MS/MS data can be structurally annotated by SIRIUS. After running the protocol, the resulting project space is about 2.5 GB in size, containing not only the dataset features but also all annotations made in the process.

Computational speed is mainly dependent on the number of aligned features in a sample, as well as the mass of the molecules. More features denote more computations to start, while feature mass increases the runtime of a single computation due to increasing computational complexity (see limitations).

## Step-by-step method details

### API connection and project initialization

**Timing: 1 min**

This step establishes communication with the SIRIUS REST service and opens or creates a project space, which is the container for all input data and annotation results.

1. Start the SIRIUS REST service. To start it in the CLI, pass the flag -s to allow shutting SIRIUS down via PySirius and the flag -p to select a custom port:

sirius rest -s --headless

# or with a custom port:

sirius rest -s -p 8080 --headless

***Optional:*** Start SIRIUS within Python by specifying the path to the executable. When using this approach, skip the next step.

from PySirius import ∗

sdk = SiriusSDK()

api = sdk.start_sirius(sirius_path=“path/to/sirius-executable”, port=8080, headless=True)

if api.actuator().health().get('status') != “UP”:

 raise RuntimeError(“SIRIUS REST service is not reachable. “

      “Check that SIRIUS started correctly.”)

***Note:*** When using the first approach, this step is the only non-Python code in this protocol.

It needs to be run on the command line where the SIRIUS executable is reachable. If needed, set the absolute path to the SIRIUS executable.

2. Import PySirius, connect to the SIRIUS service and verify that it is healthy.

from PySirius import ∗

sdk = SiriusSDK()

api = sdk.attach_or_start_sirius()

if api.actuator().health().get('status') != “UP”:

 raise RuntimeError(“SIRIUS REST service is not reachable. “

      “Check that SIRIUS started correctly.”)

***Note:*** See troubleshooting 1 if this step fails.

3. Log in to SIRIUS. Set SIRIUS_USER and SIRIUS_PW to your credentials.

accept_terms = True

account_credentials = AccountCredentials(

 username=“SIRIUS_USER”, password=“SIRIUS_PW”

)

api.account().login(accept_terms, account_credentials)

***Note:*** For registration, see the before you begin section. Running api.account().sign_up() will provide the sign-up URL and attempt to open a browser window.

**CRITICAL:** Make sure the login was successful in order to be able to use all following features.

4. Create a new, empty project space and save its information to a variable.
  project_info = api.projects().create_project (“STAR_protocol_rosemary”)

***Optional:*** If a project space already exists, open it instead. Specify the path accordingly.

project_info = api.projects().open_project(

 “STAR_protocol_rosemary”,

 “/path/to/project/file”

)

### Data import and LC-MS feature alignment

**Timing: 15 min**

Here we fetch all important data files from the rosemary dataset and group them by their metadata.

5. Set the root path to the dataset folder and collect all mzXML/mzML file paths.

root_path = “./MSV000080553”

from os import listdir

from os.path import isfile, join

folder_path = f”{root_path}/peak/Rosumarinus”

files = [

 join(folder_path, f)

 for f in listdir(folder_path)

 if isfile(join(folder_path, f)) and f.endswith((“.mzXML”, “.mzML”))

]

print(f”{len(files)} run files found”)

6. Parse the grouping of the files from the corrected group mapping file provided in the repository.

def parse_group_mapping(file_path):

 groups = {}

 with open(file_path) as f:

  for line in f:

   line = line.strip()

   if not line or '=' not in line:

    continue

   key, value = line.split('=', 1)

   groups[key] = value.split(';')

 return groups

groups = parse_group_mapping(

 f”{root_path}/updates/2026-03-25_lfnothias_1828a86c/metadata/”

 “group_mapping_rosemary_corrected.txt”

)

print(“Groups found:”, list(groups.keys()))

***Note:*** This produces multiple groupings; not all are needed to replicate the rosmarinic acid finding. The groups of interest are GROUP_YOUNG, GROUP_OLD, and GROUP_BLANKS. See troubleshooting 2 for common pitfalls in grouping.

7. Import data into SIRIUS with automated blank subtraction. Create an array of sample types mapping each file to either “Blank” or “Sample”.

from os.path import basename

sample_types = [

 “Blank” if basename(f) in groups['GROUP_BLANKS'] else “Sample”

 for f in files

]

submission_parameters = LcmsSubmissionParameters.from_dict(

 {“sampleTypes”: sample_types}

)

import_job = api.projects().import_ms_run_data_as_job(

 project_info.project_id, files, submission_parameters

)

api.wait_for_job_completion(project_info.project_id, import_job.id)

**CRITICAL:** The mapping of blank and sample runs is based on the order of the sample type list and the files list. For blank subtraction to work as intended, both lists must retain their order. See troubleshooting 3.

***Note:*** Feature alignment is done as follows: SIRIUS initially performs a preliminary alignment strictly for the purpose of recalibration. Following this step, all calibrated features are aggregated and summed to generate a mean trace across the samples. The final aligned features are then extracted directly from this mean trace. This process does not need any measured standards for calibration.

### Data quality assessment

**Timing: 1 min**

Before starting computations, inspect the quality of the dataset and filter accordingly.

8. Retrieve all aligned features.

aligned_features = api.features().get_aligned_features(

 project_info.project_id)

**Figure 1:**
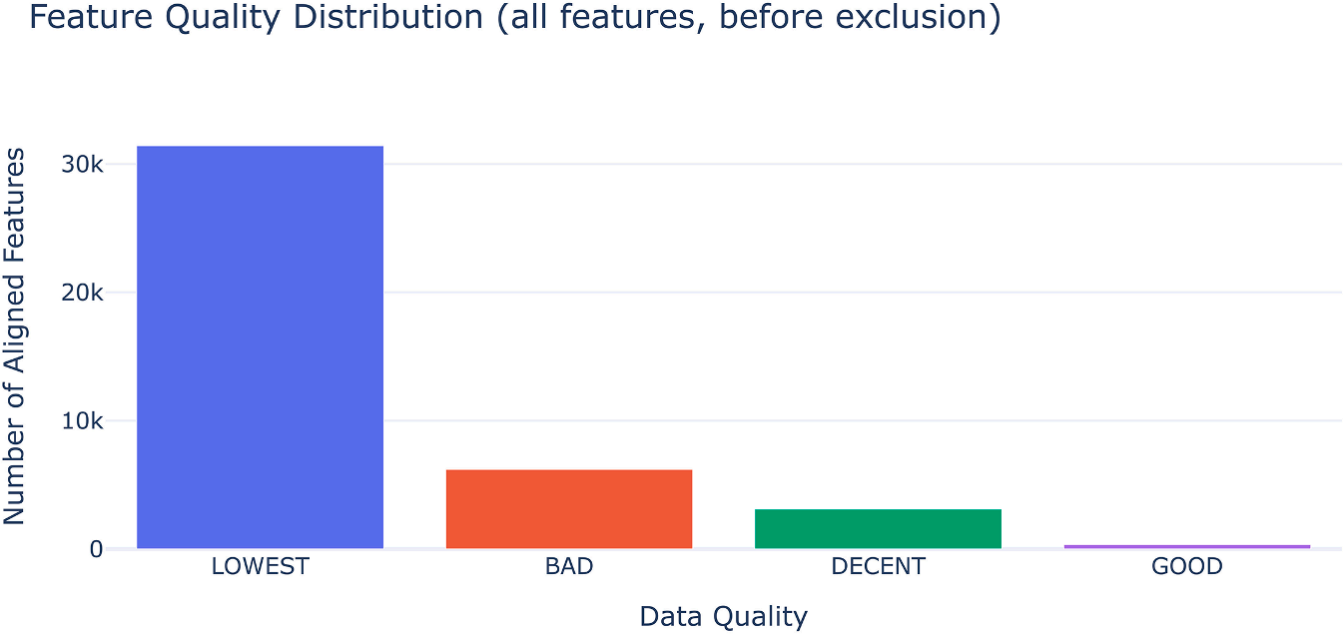
Aligned feature quality distribution histogram

9. Plot the data quality distribution. See Figure 1.

import plotly.express as px

import plotly.graph_objects as go

from collections import Counter

QUALITY_ORDER = [q.value for q in DataQuality]

palette = px.colors.qualitative.Plotly

QUALITY_COLORS = {

 quality: palette[i % len(palette)]

 for i, quality in enumerate(QUALITY_ORDER)

}

quality_counts = Counter(f.quality for f in aligned_features)

fig = go.Figure(go.Bar(

 x=QUALITY_ORDER,

 y=[quality_counts.get(k, 0) for k in QUALITY_ORDER],

 marker_color=[QUALITY_COLORS.get(k) for k in QUALITY_ORDER],

 hovertemplate=“%{x}: %{y} features<extra></extra>“

))

fig.update_layout(

 title=“Feature Quality Distribution (all features, before exclusion)”,

 xaxis_title=“Data Quality”,

 yaxis_title=“Number of Aligned Features”,

 showlegend=False,

 template=“plotly_white”

)

fig.show()

***Note:*** When applied to a whole feature, the quality measures represent a high-level summary of its suitability for an automated annotation with SIRIUS. A feature ends up with a specific overall quality based on a weighted average of its individual measures (Peak, MS/MS, Isotope, Alignment, and Adduct), followed by heuristic “cleanup” rules. The NOT_APPLICABLE data quality is comparable to NaN (Not a Number) values in numerical environments. When importing complete runs, this should not occur. When importing peaklists directly, all information about the feature quality is lost and NOT_APPLICABLE is used. See Table 1 for an explanation of data quality on features and Table 2 for a more in-depth look at what the quality levels say about the different quality measures.

10. Based on the quality distribution, exclude low-quality entries.

**Figure 2:**
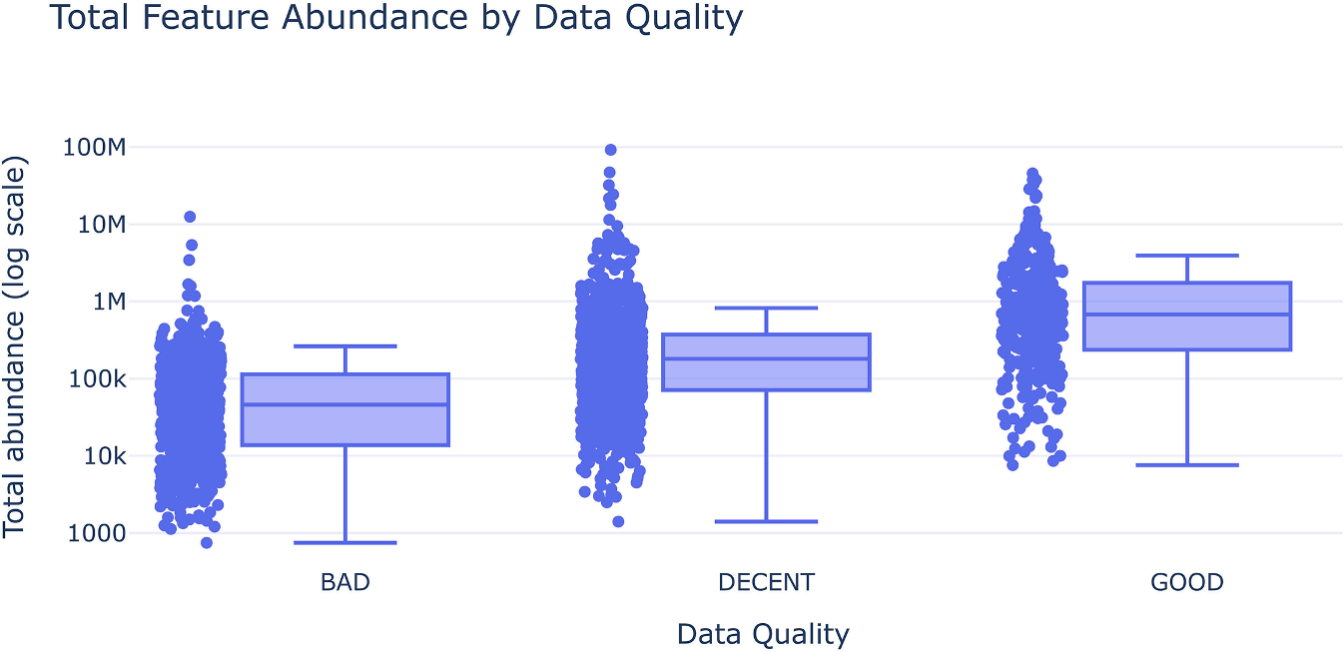
Feature abundance distribution grouped by aligned feature quality On average, the abundance of features tends to decrease with descending aligned feature qualities. Each box spans from quartile 1 (Q1; lower border) to quartile 3 (Q3; upper border). The second quartile (Q2; median) is marked by a line inside the box. The whiskers correspond to the box’ edges +/- 1.5 times the interquartile range (IQR: Q3-Q1). Note the logarithmic scale on the y-axis.

EXCLUDED_QUALITIES = [DataQuality.NOT_APPLICABLE, DataQuality.LOWEST, DataQuality.BAD]

if EXCLUDED_QUALITIES:

 aligned_features = [

  f for f in aligned_features

  if f.quality not in EXCLUDED_QUALITIES

]

 print(f”Excluded tiers: {[q.value for q in EXCLUDED_QUALITIES]}”)

else:

 print(“No quality exclusion applied.”)

print(f”{len(aligned_features)} features remaining after quality filtering”)

***Note:*** We follow the default setting in SIRIUS, including only qualities GOOD and DECENT.

***Note:*** To avoid result fluctuations through modest changes in quality filter settings (see Table 2), the SIRIUS software GUI does not allow for such changes. The presented protocol sticks with the standard SIRIUS peak quality classes.

***Note:*** We find that including lower-quality features of the rosemary dataset has little to no impact on the compound class-level fold changes produced later in the protocol. In the example of Figure 6, the same plot including features of quality BAD looks indistinguishable to the eye. The top three most abundant classes change in log2 fold change as follows (without BAD to with BAD): Flavones −1.08 to −1.19, Carotenoids −1.18 to −1.18, and Flavonols −1.46 to −1.48. All three change slightly in total abundance. This is partly due to the fact that lower-quality features tend to be less abundant; see Figure 2. Note that the lower the abundance of a class is, the more drastic additional low quality features can influence the fold change of this class.

**CRITICAL:** This filtering step shapes every downstream result. The feature IDs of the surviving features are passed directly to the SIRIUS computation job. Consequently, all fold change computations, compound annotations, and class-level statistics are based entirely on this filtered set. Features excluded here will not appear in any result, table, or plot.

**Table 1:** Summary of data quality levels for aligned features

Quality	Description
GOOD	A feature achieves this status by maintaining a high weighted average across categories and typically requiring high-quality fragmentation data for final validation.
DECENT	This level is assigned to real biological signals that achieve a moderate average score, marking them as reliable for identification despite non-ideal data characteristics.
BAD	Features fall into this category when their overall metrics are poor but they possess just enough diagnostic evidence to be kept in the data rather than discarded as noise.
LOWEST	A feature is relegated to this level if it is identified as stochastic noise or lacks the minimum combined evidence required to be considered a viable molecule for analysis.

**Table 2:** Summary of data quality levels across quality measures

Quality	Peak	Alignment	Isotopes	MS/MS	Adducts
GOOD	Strong peak; clean apex; low noise; clear edges.	Well represented; low RT error; strong consensus agreement.	Rich pattern; high trace correlation; stable ratios.	Many signal peaks; plausible deltas; low pollution.	Single known adduct.
DECENT	Usable peak; acceptable signal, shape, apex, edges.	Usable alignment; moderate RT error or weaker agreement.	Partial but useful evidence; moderate ratio stability.	Usable spectrum; limited peaks or moderate noise.	Plausible but uncertain.
BAD	Weak or questionable peak; poor shape, noise, unclear edges.	Few aligned features; high RT error; weak agreement.	Weak evidence; poor correlation or unstable ratios.	Few strong peaks; noisy; many implausible deltas.	No useful assignment.
LOWEST	Unreliable peak; below noise, ambiguous apex, undefined edges.	Failed alignment; missing or far too few features; very high RT error.	Not directly assigned; poor evidence usually maps to BAD.	Likely unusable; near-noise, charge issue, severe pollution.	Not directly assigned.

RT, retention time; apex, peak maximum; consensus trace, representative aligned peak shape; mass deltas, mass differences between MS/MS peaks; pollution, non-target ions captured in the MS/MS isolation window; adduct, observed ion form of the molecule.

### Molecular formula annotation, structure annotation, and compound class annotation

**Timing: 30 min**

This step is the most time- and resource-intensive. We annotate aligned features with molecular formulas (SIRIUS, ZODIAC^13^), structures (CSI:FingerID, COSMIC^14^) and compound classes (CANOPUS).

***Note:*** Timing varies with available cores and threads, as fragmentation trees for formula annotation are calculated locally. On a Linux compute node with two AMD EPYC 7662 64-Core Processors, totaling 256 threads, timing decreased to about 15 min.

11. Retrieve the default job configuration, optionally modify parameters, and save the configuration to the project space under a custom name.

job_submission = api.jobs().get_default_job_config(include_config_map=True)

JOB_CONFIG_NAME = “rosemary_fold_change_analysis”

api.jobs().save_job_config(JOB_CONFIG_NAME, job_submission)

***Note:*** The saved configuration is retrievable by name, making the analysis auditable and reproducible without re-submitting the full configuration object each time. Important configurable parameters include formula_id_params.profile for selecting the instrument profile (QTOF or Orbitrap), formula_id_params.mass_accuracy_ms2ppm for setting MS/MS mass accuracy, ms_novelist_params.enabled for enabling MSNovelist^15^*de novo* structural prediction, and structure_db_search_params.structure_search_dbs for restricting the CSI:FingerID database search.

12. Start a computation job from the saved configuration, passing all quality-filtered aligned feature IDs.

job = api.jobs().start_job_from_config(

 project_info.project_id,

 JOB_CONFIG_NAME,

 [f.aligned_feature_id for f in aligned_features]

)

api.wait_for_job_completion(project_info.project_id, job.id)

**CRITICAL:** Make sure the aligned features here are those of interest. In this case, we already filtered out low-quality features in the previous step. See troubleshooting 4 if no CSI:FingerID structure annotations appear after the job completes.

***Note:*** When allowing all quality levels of aligned features, we observe a clear trend toward higher annotation rates for high-quality features. See the table below.

**Table:** 

Quality level	Total al. Features	With MS/MS	Struct. Annot.	Comp. Class annot.
GOOD	362	362	343	348
DECENT	3118	1722	982	998
BAD	6208	1654	734	763
LOWEST	31438	2569	405	424
**Total**	**41126**	**6307**	**2464**	**2533**

### Sample tagging and group definition

**Timing: 1 min**

Tag runs with metadata and define named sample groups for fold change comparisons.

***Note:*** SIRIUS uses a tag-based system with Lucene query syntax to enable flexible Boolean combinations.

13. Group runs by ID.

def create_run_id_groups(runs, groups):

 name_to_id = {run.name: run.run_id for run in runs.content}

 return {

  group_name: [

   name_to_id[f.rsplit('.', 1)[0]]

   for f in filenames

   if f.rsplit('.', 1)[0] in name_to_id

 ]

  for group_name, filenames in groups.items()

 }

runs = api.runs().get_runs_page_experimental(

 project_info.project_id, size=len(files))

id_groups = create_run_id_groups(runs, groups)

***Note:*** This differs from the data import step, where only filenames were needed.

14. Define two categorical tag dimensions: plantPart (Flower/Leaf/Stem) and age (Young/Old).

plant_part_tag = TagDefinitionImport.from_dict({

 “tagName”: “plantPart”,

 “tagType”: “PROCESSING”,

 “valueType”: ValueType.TEXT,

 “possibleValues”: [“Flower”, “Leaf”, “Stem”]

})

age_tag = TagDefinitionImport.from_dict({

 “tagName”: “age”,

 “tagType”: “PROCESSING”,

 “valueType”: ValueType.TEXT,

 “possibleValues”: [“Young”, “Old”]

})

api.tags().create_tags(project_info.project_id, [plant_part_tag, age_tag])

15. Create tag submissions and attach them to runs.

tag_map = [

 ('GROUP_STEM', 'plantPart', 'Stem'),

 ('GROUP_FLOWERS', 'plantPart', 'Flower'),

 ('GROUP_LEAF', 'plantPart', 'Leaf'),

 ('GROUP_YOUNG', 'age', 'Young'),

 ('GROUP_OLD', 'age', 'Old'),

]

tag_submissions = [

 TagSubmission.from_dict({

  “tagName”: tag_name,

  “value”: value,

  “taggedObjectId”: run_id

 })

 for group_key, tag_name, value in tag_map

 for run_id in id_groups[group_key]

]

api.runs().add_tags_to_runs_experimental(

 project_info.project_id, tag_submissions

)

16. Using Lucene query syntax, define named groups for young and old leaves.

api.tags().add_group(

 project_info.project_id, “Young Leaves”,

 'tags.age:”Young” AND tags.plantPart:”Leaf”', “Samples”

)

api.tags().add_group(

 project_info.project_id, “Old Leaves”,

 'tags.age:”Old” AND tags.plantPart:”Leaf”', “Samples”

)

### Feature-level fold change analysis

**Timing: 1 min**

Compute fold changes at the aligned feature level to identify individual metabolic features that are differentially abundant between sample groups.

17. Create a fold change job submission configuration defining the left and right run groups, aggregation strategy, and quantification measure.

fold_change_submission = FoldChangeJobSubmission.from_dict({

 “leftRunGroup”: “Young Leaves”,

 “rightRunGroup”: “Old Leaves”,

 “aggregationTypes”: [AggregationType.AVG],

 “quantificationMeasures”: [QuantMeasure.AREA_UNDER_CURVE]

})

***Note:*** Multiple aggregation types (AVG, MAX, MIN) and quantification measures (AREA_UNDER_CURVE, APEX_INTENSITY) can be requested in a single submission. All requested combinations are computed in one job and retrieved independently at retrieval time.

**CRITICAL:** Ensure that runs are actually tagged and grouped before starting the fold change job. See troubleshooting 5.

18. Start the fold change computation job and wait for completion.

fc_job = api.feature_statistics()∖

 .compute_aligned_feature_fold_changes_experimental(

  project_info.project_id, fold_change_submission

 )

api.wait_for_job_completion(project_info.project_id, fc_job.id)

19. Retrieve the feature statistics table.

feature_statistics_table = api.feature_statistics()∖

 .get_aligned_feature_fold_change_table_experimental(

  project_info.project_id,

  AggregationType.AVG,

 QuantMeasure.AREA_UNDER_CURVE

 )

### Verification of a known hypothesis: Replicating the rosmarinic acid finding

**Timing: 1 min**

Up to this point, the analysis was carried out in a completely untargeted fashion, not concentrating on any particular compounds or compound classes known beforehand. To demonstrate that we can also provide insights for a more targeted setting, we will now concentrate on a single compound, replicating established knowledge that rosmarinic acid is present at different abundances in leaves of different ages.^9^^,^^10^^,^^11^

20. Resolve the PubChem CID for rosmarinic acid programmatically.

import pubchempy as pcp

results = pcp.get_compounds('Rosmarinic acid', 'name')

if not results:

 raise RuntimeError(“Rosmarinic acid not found in PubChem.”)

rosmarinic_acid_pubchem_id = results[0].cid

print(f”PubChem CID for rosmarinic acid: {rosmarinic_acid_pubchem_id}”)

21. Retrieve aligned features once more, now with their top annotations.

aligned_features = api.features().get_aligned_features(

 project_info.project_id,

 opt_fields=[AlignedFeatureOptField.TOPANNOTATIONS]

)

***Note:*** This list now again includes all aligned features, not just the quality-filtered subset. All following steps specifically ask for features with annotations, which, because only the features in the subset were calculated, will include at most that filtered subset.

22. Filter aligned features for those annotated with the retrieved PubChem CID.

pubchem_db_link = DBLink.from_dict({

 'name': 'PUBCHEM',

 'id': str(rosmarinic_acid_pubchem_id)

})

rosmarinic_acid_features = [

 f for f in aligned_features

 if f.top_annotations.structure_annotation is not None

 and pubchem_db_link in f.top_annotations.structure_annotation.db_links

]

23. Select the protonated molecule [M+H]^+^ as the primary feature.

rosmarinic_acid_base_feature = next(

 f for f in rosmarinic_acid_features

 if f.top_annotations.formula_annotation.adduct == '[M + H]+'

)

***Note:*** Multiple hits are expected when the same compound is detected as different adduct ions. The [M+H]^+^ adduct is selected as it is the most commonly observed adduct in positive-mode ESI.

***Note:*** Putting very strict quality constraints on the aligned features can lead to the loss of many features. The [M+H]^+^ adduct ion for rosmarinic acid is of data quality DECENT, and filtering to keep only GOOD features would make this analysis fail. See troubleshooting 6.

24. Handle zero-value fold-changes.

***Note:*** Entries absent in the left group but present in the right group need special handling. They produce a fold change value of 0, which is undefined in log2 space. Set them to negative infinity to keep them complementary to positive infinity values arising from the inverse case.

import math

def log2_fold_change(raw_fc, invert=False):

 “““Return a signed log2 fold change while preserving zero as infinity.

 A raw fold change of 0 means the left group is absent and the right group is present, so log2(left / right) is -inf. Negative fold changes are invalid.

 “““

 if raw_fc < 0:

  raise ValueError(f”Fold change ratios cannot be negative: {raw_fc}”)

 if raw_fc == 0:

  log2_fc = -math.inf

 else:

  log2_fc = math.log2(raw_fc)

 return -log2_fc if invert else log2_fc

def displayed_fold_change(raw_fc, invert=False):

 “““Return the fold change ratio in the displayed group order.”““

 if raw_fc < 0:

  raise ValueError(f”Fold change ratios cannot be negative: {raw_fc}”)

 if not invert:

  return raw_fc

 return math.inf if raw_fc == 0 else 1 / raw_fc

25. Define a helper function to extract the log2 fold change for a specific feature and group pairing.

def get_fold_change(statistics_table, row_id, group1, group2):

 “““Return the log2 fold change of group1 relative to group2 for a given feature.”““

 try:

  row_idx = statistics_table.row_ids.index(row_id)

 except ValueError:

  return None

 for col_idx, (left, right) in enumerate(zip(

  statistics_table.column_left_groups,

  statistics_table.column_right_groups

 )):

  if left == group1 and right == group2:

   fc = statistics_table.values[row_idx][col_idx]

   return log2_fold_change(fc)

  elif left == group2 and right == group1:

   fc = statistics_table.values[row_idx][col_idx]

   return log2_fold_change(fc, invert=True)

 return None

26. Execute the function to compute the fold change of rosmarinic acid between old and young leaves.

log2_fc = get_fold_change(

  feature_statistics_table,

  rosmarinic_acid_base_feature.aligned_feature_id,

  “Old Leaves”, “Young Leaves”

)

print(f”Rosmarinic acid log2(Old / Young) = {log2_fc:.3f} “

  f”(fold change = {2∗∗log2_fc:.2f}x)”)

***Note:*** A positive log2 fold change confirms that rosmarinic acid is more abundant in old leaf tissue, replicating the prior known findings.^9^^,^^10^^,^^11^ Running this analysis, the result is a log2 fold change of 2.462, corresponding to more than a 5.51-fold enrichment in old leaves. In an untargeted approach, this check supports a deeper investigation of the rosmarinic acid feature.

### Highest differentially abundant features analysis

**Timing: 1 min**

This step ranks all features that received a structural annotation from CSI:FingerID by the absolute magnitude of their log2 fold change.

27. Define a function to construct a DataFrame of annotated fold changes.

***Note:*** Infinite fold change values are retained. Zero-valued fold changes are set as negative infinity log2 fold changes.

import pandas as pd

import numpy as np

def get_annotated_fold_changes_df(statistics_table, group1, group2, aligned_features):

 “““Return a DataFrame of log2 fold changes for all CSI:FingerID-annotated features, ordered by absolute log2 fold change (descending).”““

 col_idx, invert = None, False

 for idx, (left, right) in enumerate(zip(

  statistics_table.column_left_groups,

  statistics_table.column_right_groups

 )):

  if left == group1 and right == group2:

   col_idx = idx; break

  elif left == group2 and right == group1:

   col_idx = idx; invert = True; break

 if col_idx is None:

  return pd.DataFrame()

 feature_meta = {}

 for f in aligned_features:

  if f.top_annotations and f.top_annotations.structure_annotation is not None:

   name = f.top_annotations.structure_annotation.structure_name or ““

   feature_meta[f.aligned_feature_id] = (f.quality, name)

 rows = []

 for row_idx, feature_id in enumerate(statistics_table.row_ids):

  if feature_id not in feature_meta:

   continue

  raw = statistics_table.values[row_idx][col_idx]

  log2_fc = log2_fold_change(raw, invert=invert)

  quality, compound_name = feature_meta[feature_id]

  left_abundance = statistics_table.left_abundances[row_idx][col_idx]

  right_abundance = statistics_table.right_abundances[row_idx][col_idx]

  rows.append({

   'feature_id': feature_id,

   'compound_name': compound_name,

   'quality': quality,

   'log2_fc': log2_fc,

   'fold_change': displayed_fold_change(raw, invert=invert),

   'direction': f”{group1}/{group2}”,

   'leftAbundance': left_abundance,

   'rightAbundance': right_abundance,

  })

  return (

   pd.DataFrame(rows)

   .assign(abs_log2_fc=lambda d: d['log2_fc'].abs())

   .sort_values('abs_log2_fc', ascending=False)

   .drop(columns='abs_log2_fc')

   .reset_index(drop=True)

  )

28. Execute the function for the Young Leaves vs. Old Leaves comparison.

fc_df = get_annotated_fold_changes_df(

 feature_statistics_table, “Young Leaves”, “Old Leaves”, aligned_features

)

print(f”{len(fc_df)} annotated features with fold change values, including zero and infinite values”)

***Optional:*** To replicate the plot from Figure 2, run the following code after creating fc_df.

import plotly.express as px

fc_df[“totalAbundance”] = fc_df[“leftAbundance”].fillna(0) + fc_df[“rightAbundance”].fillna(0)

plot_df = fc_df[

 fc_df[“totalAbundance”].notna()

 ∖& (fc_df[“totalAbundance”] > 0)

].copy()

plot_df[“quality”] = plot_df[“quality”].astype(str)

fig = px.box(

 plot_df,

 x=“quality”,

 y=“totalAbundance”,

 points=“all”,

 category_orders={“quality”: [str(q.value) for q in DataQuality]},

 hover_data=[“feature_id”, “compound_name”, “log2_fc”],

 log_y=True,

 title=“Total Feature Abundance by Data Quality”

)

fig.update_layout(

 xaxis_title=“Data Quality”,

 yaxis_title=“Total abundance (log scale)”,

 template=“plotly_white”

)

fig.show()

29. If only interested in up- or downregulation, filter out infinite values. Then, inspect the top-ranking candidate.

fc_df_finite = fc_df[np.isfinite(fc_df['log2_fc'])]

if fc_df_finite.empty:

 raise RuntimeError(“No annotated feature with finite fold change found.”)

top_row = fc_df_finite.iloc[0]

top_id = top_row['feature_id']

top_feature = api.features().get_aligned_feature(

 project_info.project_id, top_id,

 opt_fields=[AlignedFeatureOptField.TOPANNOTATIONS]

)

print(f”Highest FC feature quality:    {top_feature.quality}”)

print(f”Log2 fold change:      {top_row['log2_fc']}”)

print(f”Structure annotation: {top_feature.top_annotations.structure_annotation.structure_name}”)

***Note:*** When allowing lower-quality features to pass through the quality filtering, a BAD or LOWEST quality feature at the top of the ranking should be interpreted with caution.

***Optional:*** Apply an additional post hoc filter to retain only GOOD quality features without re-running the computation job.

fc_df_high_quality = fc_df_finite[fc_df_finite['quality'].isin([DataQuality.GOOD])]

***Optional:*** When specifically looking for features not expressed in the right class but expressed in the left class, filter for infinite values and inspect the entries.

fc_df_infinite = fc_df[np.isinf(fc_df['log2_fc'])]

**CRITICAL:** Fold changes are direction-dependent. Features absent in the left class and present in the right class will have a fold change of 0. To obtain these features with positive infinity values, switch the order of the classes. Note again that we artificially set fold changes of 0 to a log2 fold change of negative infinity to minimize this effect.

### Compound class-level fold change analysis

**Timing: 1 min**

Aggregate abundances at the level of compound classes to obtain a robust overview of which biosynthetic pathways or chemical families are differentially represented. SIRIUS computes class fold changes from the actual summed abundance of annotated features within each class, not by counting annotations.

30. Submit compound class fold change jobs

***Note:*** You can choose between both the Natural Products Classifier (NPC)^16^ and ClassyFire^17^ hierarchies.

compound_class_fold_change_submission = FoldChangeJobSubmission.from_dict({

 “leftRunGroup”: “Young Leaves”,

 “rightRunGroup”: “Old Leaves”,

 “aggregationTypes”: [AggregationType.AVG],

 “quantificationMeasures”: [QuantMeasure.AREA_UNDER_CURVE]

})

npc_fc_job = api.npc_class_statistics()∖

 .compute_npc_class_fold_changes_experimental(

  project_info.project_id, compound_class_fold_change_submission

)

api.wait_for_job_completion(project_info.project_id, npc_fc_job.id)

classyfire_fc_job = api.classyfire_class_statistics()∖

 .compute_classyfire_class_fold_changes_experimental(

  project_info.project_id, compound_class_fold_change_submission

)

api.wait_for_job_completion(project_info.project_id, classyfire_fc_job.id)

31. Retrieve the statistics tables for NPC and ClassyFire.

npc_statistics_table = api.npc_class_statistics()∖

 .get_npc_class_fold_change_table_experimental(

  project_info.project_id,

  aggregation=AggregationType.AVG,

  quantification=QuantMeasure.AREA_UNDER_CURVE

 )

classyfire_statistics_table = api.classyfire_class_statistics()∖

 .get_classyfire_class_fold_change_table_experimental(

  project_info.project_id,

  aggregation=AggregationType.AVG,

  quantification=QuantMeasure.AREA_UNDER_CURVE

 )

32. Convert the raw tables into Pandas DataFrames and execute the parsing function on both tables.

def parse_class_statistics_table(statistics_table, group1, group2):

 “““Convert a class-level statistics table to a DataFrame.”““

 col_idx, invert = None, False

 for idx, (left, right) in enumerate(zip(

  statistics_table.column_left_groups,

  statistics_table.column_right_groups

 )):

  if left == group1 and right == group2:

   col_idx = idx; break

  elif left == group2 and right == group1:

   col_idx = idx; invert = True; break

 assert col_idx is not None, f”Comparison {group1} vs {group2} not found.”

 rows = []

 for row_idx, row_id in enumerate(statistics_table.row_ids):

  left_abundance = statistics_table.left_abundances[row_idx][col_idx]

  right_abundance = statistics_table.right_abundances[row_idx][col_idx]

  raw = statistics_table.values[row_idx][col_idx]

  log2_fc = log2_fold_change(raw, invert=invert)

  total_ab = (left_abundance or 0) + (right_abundance or 0)

  rows.append({

   “row_id”: row_id,

   “name”: statistics_table.row_names[row_idx],

   “level”: statistics_table.row_levels[row_idx],

   “log2_fc”: log2_fc,

   “abs_log2_fc”: abs(log2_fc),

   “enriched_in”: group1 if log2_fc > 0 else group2,

   “leftAbundance”: left_abundance,

   “rightAbundance”: right_abundance,

   “totalAbundance”: total_ab,

  })

 df = pd.DataFrame(rows).sort_values(“abs_log2_fc”, ascending=False)

 print(f”{len(df)} class entries retrieved.”)

 return df

npc_df = parse_class_statistics_table(

 npc_statistics_table, “Young Leaves”, “Old Leaves”

)

classyfire_df = parse_class_statistics_table(

 classyfire_statistics_table, “Young Leaves”, “Old Leaves”

)

33. Define visualization utility parameters and a filtering function.

ABS_LOG2_FC_THRESHOLD = 1

COLOR_GROUP1 = “#378ADD”

COLOR_GROUP2 = “#D85A30”

def _filter(df, threshold, levels):

 mask = df[“abs_log2_fc”] >= threshold

 if levels is not None:

  mask &= df[“level”].isin(levels)

 return df[mask].copy()

def _with_plot_log2_fc(df):

 “““Add finite plot_log2_fc values while preserving true log2_fc values.”““

 sub = df.copy()

 finite = sub[np.isfinite(sub[“log2_fc”])]

 if finite.empty:

  replacement = 1.0

 else:

  replacement = 2 ∗ finite[“log2_fc”].abs().max()

 sub[“plot_log2_fc”] = sub[“log2_fc”].where(

  np.isfinite(sub[“log2_fc”]),

  np.sign(sub[“log2_fc”]) ∗ replacement,

 )

 return sub

def _title(group1, group2, levels, threshold):

 lvl_str = “, “.join(levels) if levels else “all levels”

 return f”{group1} vs {group2} | {lvl_str} | |log2 FC| >= {threshold}”

34. Define a plotting function for raw group abundance sunbursts and inspect the absolute class abundances for each group before examining fold changes.

def plot_hierarchical_abundance_sunbursts(classyfire_df, cf_hierarchy, group1, group2, max_levels=-1):

 “““

 Plots a dual sunburst chart for hierarchical abundances.

 Parameters:

 - max_levels (int): Maximum number of hierarchical levels to show initially.

     Default is -1 (shows all levels). Setting it to 2 shows the root and 1 outer ring.

 “““

 id_to_name = cf_hierarchy.set_index(“id”)[“name”].to_dict()

 id_to_parent = cf_hierarchy.set_index(“id”)[“parentId”].to_dict()

 name_to_id = {v: k for k, v in id_to_name.items()}

 sub = classyfire_df.dropna(subset=[“leftAbundance”, “rightAbundance”]).copy()

 sub = sub[sub[“totalAbundance”] > 0]

 sub[“chemont_id”] = sub[“name”].map(name_to_id)

 sub = sub.dropna(subset=[“chemont_id”])

 def get_ancestors(cid):

  ancestors, current = [], id_to_parent.get(cid)

  while current and not pd.isna(current):

   ancestors.append(current)

   current = id_to_parent.get(current)

  return ancestors

 all_ids = set(sub[“chemont_id”])

 for cid in list(all_ids):

  all_ids.update(get_ancestors(cid))

 leaf_left = sub.set_index(“chemont_id”)[“leftAbundance”].to_dict()

 leaf_right = sub.set_index(“chemont_id”)[“rightAbundance”].to_dict()

 nodes = []

 for cid in all_ids:

  parent = id_to_parent.get(cid)

  nodes.append({

   “id”: cid,

   “label”: id_to_name.get(cid, cid),

   “parent”: parent if parent and not pd.isna(parent) else ““,

   “left”: leaf_left.get(cid, 0),

   “right”: leaf_right.get(cid, 0),

  })

 nodes_df = pd.DataFrame(nodes)

 # drop trivial “chemical entities” class

 root_ids = nodes_df[nodes_df[“parent”] == ““][“id”].tolist()

 nodes_df = nodes_df[∼nodes_df[“id”].isin(root_ids)].copy()

 nodes_df.loc[nodes_df[“parent”].isin(root_ids), “parent”] = ““

 fig = go.Figure()

 for values_col, colorscale, domain, name in [

  (“left”, “Blues”, [0, 0.48], group1),

  (“right”, “Oranges”, [0.52, 1.0], group2),

 ]:

  fig.add_trace(go.Sunburst(

   ids=nodes_df[“id”],

   labels=nodes_df[“label”],

   parents=nodes_df[“parent”],

   values=nodes_df[values_col],

   name=name,

   domain={“x”: domain},

   marker_colorscale=colorscale,

   branchvalues=“remainder”,

   maxdepth=max_levels,

   hovertemplate=“<br>%{label}</br><br>Abundance: %{value:.2e}<extra></extra>”,

  ))

 fig.update_layout(

  title=f”ClassyFire Hierarchical Class Abundance: {group1} (left) vs {group2} (right)”,

  annotations=[

   dict(text=group1, x=0.20, y=1.08, font_size=13, showarrow=False, xref=“paper”, yref=“paper”),

   dict(text=group2, x=0.80, y=1.08, font_size=13, showarrow=False, xref=“paper”, yref=“paper”),

  ],

  height=700, template=“plotly_white”,

  margin=dict(t=100, b=40, l=40, r=40),

 )

 fig.show()

***Note:*** Examine the raw abundance sunbursts before the fold change sunburst. Slice sizes within a single panel reflect the absolute contribution of each class in that group, but sizes cannot be compared directly across the two panels because the total abundance summed over all classes may differ substantially between groups.

**Figure 3:**
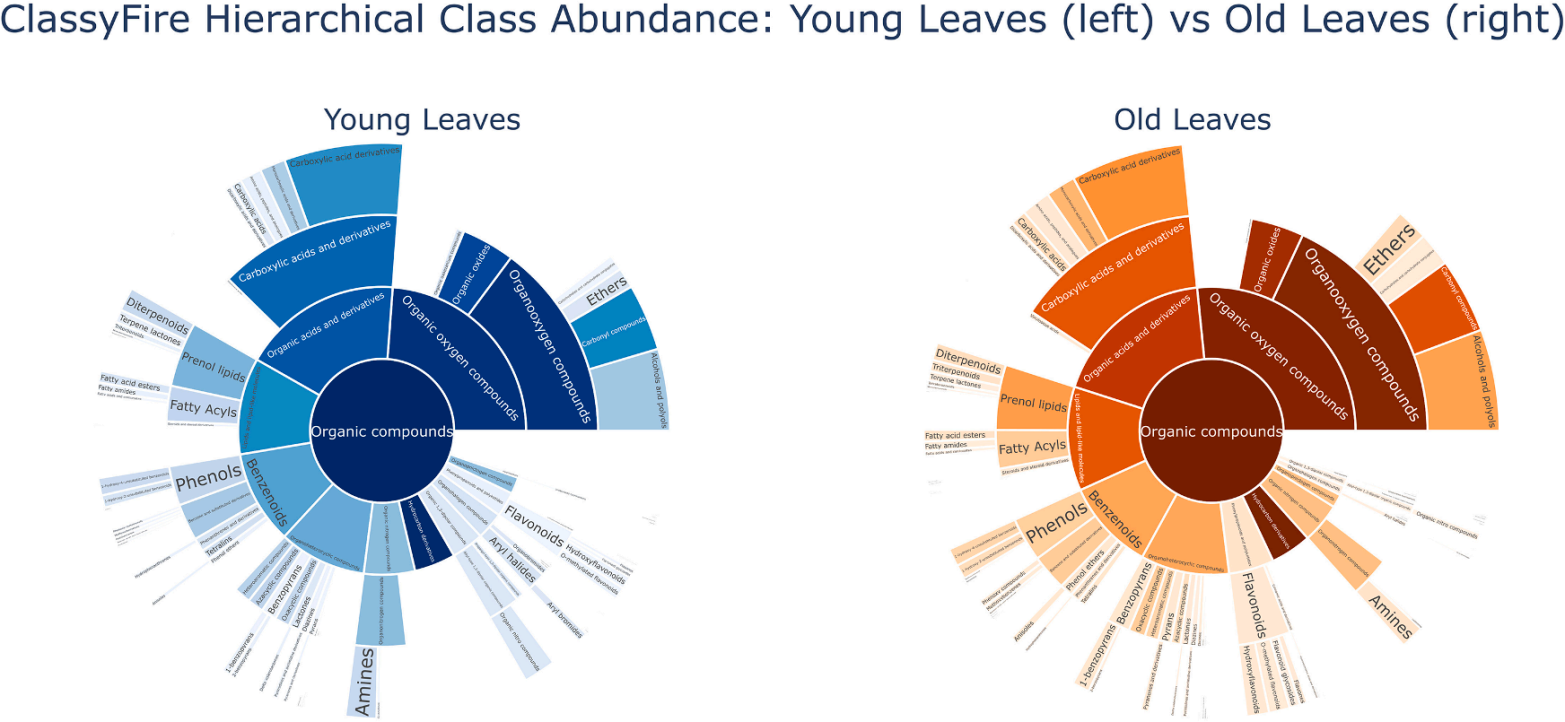
Sunburst plots for comparing the abundance of ClassyFire classes predicted by CANOPUS in two groups The layering of classes follows the ClassyFire hierarchy. The color encodes abundance (dark, high abundance; light, low abundance). Here, a depth of four levels in the hierarchy is chosen for visualization.

35. Generate raw abundance sunbursts per NPC hierarchy level. For this, fetch the ClassyFire hierarchy mapping. See Figure 3.

from io import StringIO

classyfire_hierarchy = api.projects().get_canopus_classy_fire_data(project_info.project_id, 1)

classyfire_hierarchy = pd.read_csv(StringIO(classyfire_hierarchy), sep=“∖t”)

plot_hierarchical_abundance_sunbursts(classyfire_df, classyfire_hierarchy, “Young Leaves”, “Old Leaves”, 4)

**CRITICAL:** Annotation requirements are low, especially for CANOPUS compound class annotation, which only requires that the MS/MS spectrum contains at least two peaks besides the molecular ion peak. Even then, this is clearly not the entire metabolome of our sample. Moreover, our annotations are restricted by the technology employed, as we also cannot annotate compounds that do not ionize, separate, or fragment well. The decision to include only features of at least decent quality in the analysis is made to prevent low-quality features from dominating the reported statistics.

36. Define plotting functions for bar charts, strip plots, and single-level sunburst charts.

def plot_bar(df, group1, group2, threshold=ABS_LOG2_FC_THRESHOLD, levels=None):

 sub = _with_plot_log2_fc(_filter(df, threshold, levels))

 colors = [COLOR_GROUP1 if v > 0 else COLOR_GROUP2 for v in sub[“log2_fc”]]

 fig = go.Figure(go.Bar(

  x=sub[“plot_log2_fc”], y=sub[“name”],

  orientation=“h”,

  marker_color=colors,

  customdata=sub[“log2_fc”],

  hovertemplate=“%{y}<br>log2 FC: %{customdata:.2f}<br>plotted as: %{x:.2f}<extra></extra>“

 ))

 fig.update_layout(

  title=_title(group1, group2, levels, threshold),

  xaxis_title=“log2 FC (infinite values plotted at 2x max finite |log2 FC|)”,

  xaxis=dict(zeroline=True, zerolinecolor=“black”, zerolinewidth=1),

  height=max(400, len(sub) ∗ 20),

  template=“plotly_white”

 )

 fig.show()

def plot_strip(df, group1, group2, threshold=ABS_LOG2_FC_THRESHOLD, levels=None):

 sub = _with_plot_log2_fc(_filter(df, threshold, levels))

 fig = px.strip(

  sub, x=“plot_log2_fc”, y=“level”,

  color=“enriched_in”,

  hover_name=“name”,

  hover_data={“log2_fc”: “:.2f”, “plot_log2_fc”: “:.2f”},

  color_discrete_map={group1: COLOR_GROUP1, group2: COLOR_GROUP2},

  category_orders={“level”: sorted(sub[“level”].unique())},

  title=_title(group1, group2, levels, threshold)

 )

 fig.update_layout(

  xaxis_title=“log2 FC (infinite values plotted at 2x max finite |log2 FC|)”,

  xaxis=dict(zeroline=True, zerolinecolor=“black”, zerolinewidth=1),

  template=“plotly_white”

 )

 fig.show()

def plot_sunburst(df, group1, group2, threshold=ABS_LOG2_FC_THRESHOLD, levels=None):

 “““Sunburst where color encodes log2 fold change and slice size encodes total class abundance.”””

 sub = _with_plot_log2_fc(_filter(df, threshold, levels))

 fig = px.sunburst(

  sub, path=[“level”, “name”],

  values=“totalAbundance”,

  color=“plot_log2_fc”,

  color_continuous_scale=“RdBu”,

  color_continuous_midpoint=0,

  hover_data={“log2_fc”: “:.2f”, “enriched_in”: True, “leftAbundance”: True, “rightAbundance”: True},

  title=_title(group1, group2, levels, threshold)

 )

 fig.update_coloraxes(colorbar_title=“log2 FC plotted”)

 fig.update_layout(

  template=“plotly_white”

 )

 fig.show()

**Figure 4:**
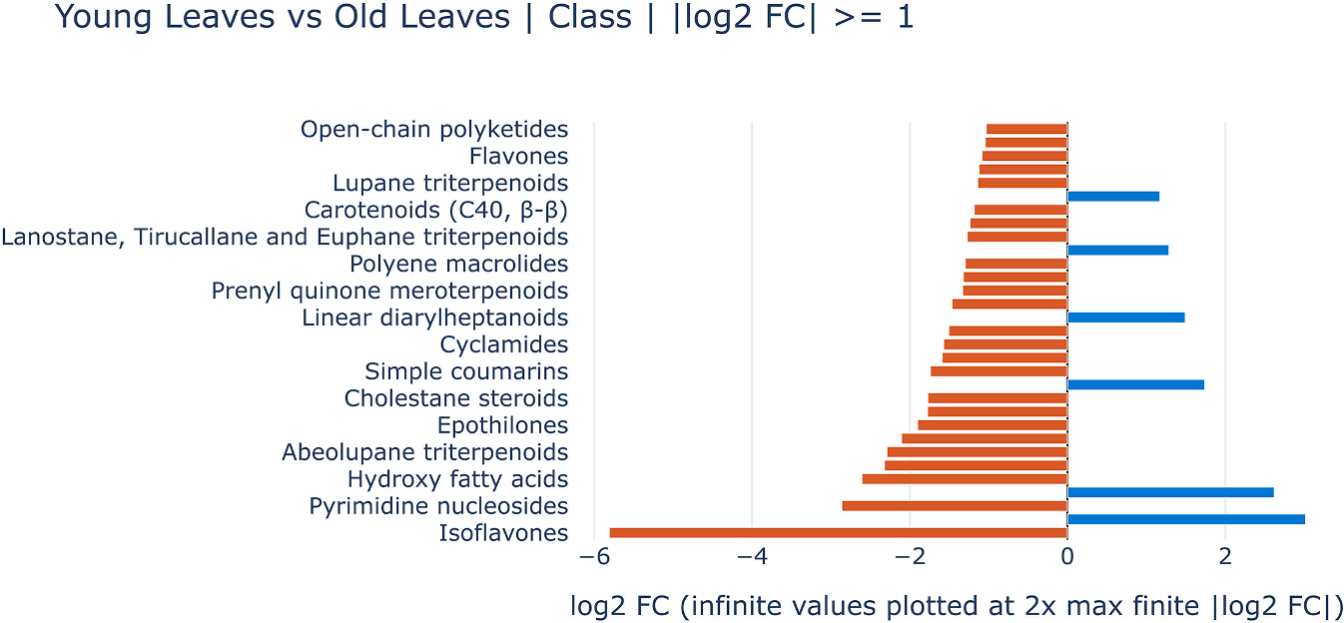
Bar plot comparing fold changes of NPC classes in two groups Threshold set to a minimum log2 fold change of 1.

**Figure 5:**
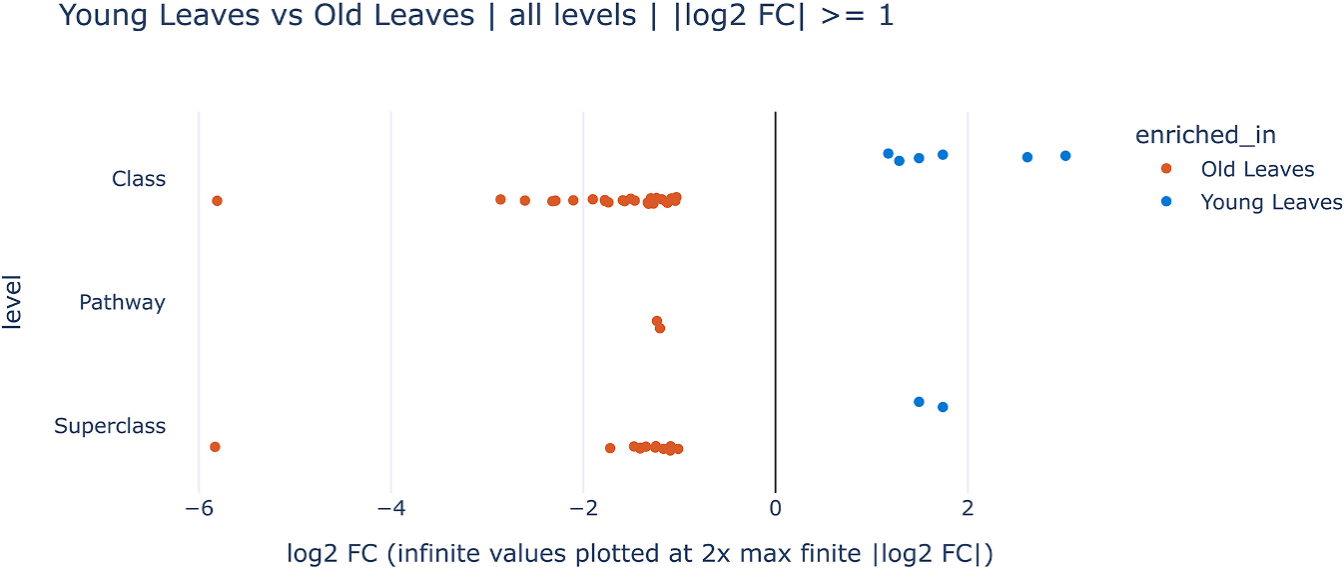
Strip plot comparing fold changes of all NPC levels in two groups Threshold set to a minimum log2 fold change of 1.

**Figure 6:**
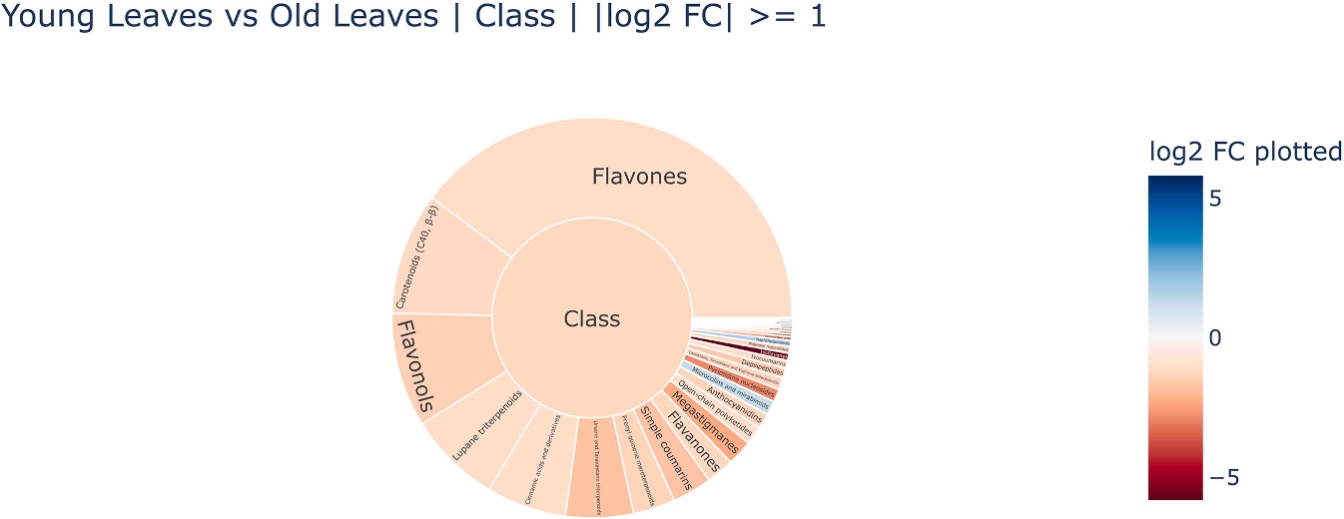
Sunburst plot comparing fold changes of NPC classes in two groups The color encodes fold change (dark blue, high positive log2 fold change; dark red, high negative log2 fold change), while the size of the slices encodes total abundance (shared over both groups). Threshold set to a minimum log2 fold change of 1.

37. Generate bar charts per hierarchy level, strip plots, and sunburst charts for NPC. Repeat for ClassyFire as needed. See Figures 4, 5, and 6.

for level in npc_df[“level”].unique():

 plot_bar(npc_df, “Young Leaves”, “Old Leaves”, levels=[level])

plot_strip(npc_df, “Young Leaves”, “Old Leaves”)

for level in npc_df[“level”].unique():

 plot_sunburst(npc_df, “Young Leaves”, “Old Leaves”, levels=[level])

# ClassyFire bar charts:

for level in classyfire_df[“level”].unique():

 plot_bar(classyfire_df, “Young Leaves”, “Old Leaves”, levels=[level])

***Note:*** These visualizations are illustrative starting points. The most informative statistics and plots depend on the specific dataset and research question. The DataFrames npc_df, classyfire_df, and fc_df as well as the raw statistics tables can be used as starting points for further analysis. See troubleshooting 7 if very few classes appear above the threshold.

### Cross-referencing feature and compound class trends

**Timing: 1 min**

This step verifies whether the compound classes annotated for rosmarinic acid follow the same enrichment trend as the feature itself.

38. Retrieve the CANOPUS compound class annotations for the rosmarinic acid base feature.

canopus_result = api.features().get_canopus_prediction(

 project_info.project_id,

 rosmarinic_acid_base_feature.aligned_feature_id,

 rosmarinic_acid_base_feature.top_annotations.formula_annotation

  .formula_id

)

39. Extract annotated NPC and ClassyFire classes with probability > 50% and compare their enrichment direction.

ros_npc_classes = [

 c.name for c in canopus_result.npc_classes if c.probability >= 0.5

]

npc_lookup = npc_df.set_index('name')['enriched_in'].to_dict()

for cls_name in ros_npc_classes:

 direction = npc_lookup.get(cls_name)

 if direction is None:

  print(f” {cls_name}: not found in NPC fold change table”)

 elif direction == “Young Leaves”:

  print(f” {cls_name}: ∗∗∗ enriched in YOUNG Leaves (counter-trend!) ∗∗∗”)

 else:

  print(f” {cls_name}: enriched in Old Leaves (consistent)”)

40. Repeat the lookup for ClassyFire classes using classyfire_df.

ros_classyfire_classes = [

 c.name for c in canopus_result.classy_fire_classes

 if c.probability >= 0.5

]

classyfire_lookup = classyfire_df.set_index('name')['enriched_in'].to_dict()

for cls_name in ros_classyfire_classes:

 direction = classyfire_lookup.get(cls_name)

 if direction is None:

  print(f” {cls_name}: not found in ClassyFire fold change table”)

 elif direction == “Young Leaves”:

  print(f” {cls_name}: ∗∗∗ enriched in YOUNG Leaves (counter-trend!) ∗∗∗”)

 else:

  print(f” {cls_name}: enriched in Old Leaves (consistent)”)

***Note:*** A counter-trend class warrants a closer look at which other features may be driving that class's overall abundance. We have found no such trend. In an untargeted approach, this check supports a deeper investigation of the rosmarinic acid feature.

### Cleanup

**Timing: 1 min**

Release file handles and shut down the REST service when the analysis is complete.

41. Close the project space.
  api.projects().close_project(project_info.project_id)

***Note:*** All data, annotations, and tables remain saved in the.sirius project file.

42. Shut down SIRIUS.
  sdk.shutdown_sirius()

***Note:*** If SIRIUS is shut down manually beforehand, this can produce an error in the SDK's shutdown function. In this case, the error may be ignored.

## Expected outcomes

Execution of this protocol yields a comprehensive, self-contained programmatic analysis of LC-MS/MS metabolomics data. At the feature level, it produces a table (e.g., a Pandas DataFrame) containing annotated features ranked by the absolute magnitude of their log2 fold change, along with their SIRIUS DataQuality flag, structural annotation (e.g., via CSI:FingerID), and absolute abundances. As demonstrated in the validation task, users should successfully replicate the finding that specific targets, such as rosmarinic acid, are significantly more abundant in specific sample cohorts (e.g., old leaves).

At the compound class level, the protocol produces hierarchical enrichment data based on both the Natural Products Classifier (NPC) and ClassyFire ontologies. Expected outputs include DataFrames that detail class enrichment directions, total abundances, and fold changes. Using the provided plotting utilities, one can generate bar charts, multi-level strip plots, and hierarchical sunburst plots.

## Quantification and statistical analysis

### Data quality filtering

Inclusion/exclusion criteria are strictly defined prior to computation. Aligned features with quality flags GOOD or DECENT are kept, therefore excluding those with qualities BAD and LOWEST which suggest subpar data quality. Because formula annotation, structure annotation, and class annotation run exclusively on this filtered set, all downstream fold change computations and statistics are grounded entirely in high-confidence data.

### Fold change aggregation

SIRIUS handles fold change quantification internally. Ratios are calculated using aggregated feature abundances between two defined tag groups (e.g., Young vs. Old).

### Class-level computations

Compound class fold changes are mathematically derived from the actual summed abundance of the annotated features within each class, providing a more robust metric than simple annotation counting.

### Infinite values

If a feature is present in the left group but completely absent in the right one, SIRIUS assigns a fold change of Double.POSITIVE_INFINITY. To fit these values into the plots, we apply an ad hoc adjustment: they are retained in the DataFrames and set to an arbitrary ceiling of twice the maximum absolute finite fold change. In inverse cases, where a feature is absent in the left group but present in the right one, a fold change of zero is calculated. We translate them into negative infinity log2 fold changes to allow those values to be highlighted as strongly as positive infinity.

## Limitations

While this protocol provides a streamlined programmatic workflow for metabolomics fold-change analysis, it does have a few notable limitations. First, the molecular formula annotation, structure annotation, and compound class annotation steps are highly resource-intensive and may require several hours to complete, depending on the dataset size and available hardware. Running time is strongly affected by the masses of annotated compounds (precursor masses of features); molecular formula annotation requires substantially more running time if compounds are large.^2^ PySirius requires an active SIRIUS REST service with internet access for CSI:FingerID and CANOPUS annotations. Offline operation is not supported for these annotation tools. Second, the accuracy and reliability of the entire fold-change analysis are heavily dependent on the initial DataQuality filtering threshold applied before the computation jobs. Excluding too many quality tiers may result in the loss of valid biological signals, whereas retaining LOWEST or BAD quality features can artificially inflate fold-change magnitudes due to sparse or noisy detection across sample replicates. Third, the compound class-level fold changes are restricted by annotation coverage: they only account for features that successfully received a class annotation via CANOPUS, meaning unannotated features are inherently excluded from these specific class-level aggregations. The quality of compound class annotations differs depending on the compound class itself; see the original publication^5^ for all statistics. Finally, SIRIUS preprocessing is currently limited to LC-MS/MS spectra. Other data, such as ion mobility data, have to be preprocessed using other software^18^^,^^19^ before performing annotations using SIRIUS.

## Troubleshooting

### Problem 1

The client cannot connect to SIRIUS. Related to Step 1.

### Potential solution

If SIRIUS is already running, attach to it manually with

sdk.attach_to_sirius(sirius_major_version=6, port=PORT_NUMBER)

using the same port as specified with -p PORT_NUMBER at startup. If the SIRIUS executable is not on the system path, launch it directly from Python with

sdk.start_sirius(sirius_path=SIRIUS_PATH, port=PORT_NUMBER)

instead of starting it on the command line. For any issue with SIRIUS not being found, try setting the absolute path to the executable file.

### Problem 2

Sample runs are assigned to mutually exclusive sample groups simultaneously (e.g., a file belongs to both “Young” and “Old” categories). This logical impossibility often occurs due to substring collisions when metadata mapping files are generated automatically. For example, in the reference rosemary dataset, a text search for the developmental zone “Z1” captures the “Z10” files, incorrectly placing Z10 runs into both the Young (Z1–Z5) and Old (Z6–Z10) sample groups. Related to Step 6.

### Potential solution

Load the original (uncorrected) mapping file and diagnose the overlapping groups with the following code.

groups_buggy = parse_group_mapping(

 f”{root_path}/other/group_mapping_rosemary.txt”

)

def are_groups_disjoint(∗group_keys, groups):

 intersection = set(groups[group_keys[0]])

 for key in group_keys[1:]:

  intersection &= set(groups[key])

 return len(intersection) == 0

def report_group_intersections(∗group_keys, groups):

 value_to_groups = {}

 for key in group_keys:

  for value in groups[key]:

   value_to_groups.setdefault(value, []).append(key)

  return {v: g for v, g in value_to_groups.items() if len(g) > 1}

print(“Blanks ∩ Samples = ∅ :”, are_groups_disjoint(

 'GROUP_BLANKS', 'GROUP_SAMPLES', groups=groups_buggy))

print(“Young ∩ Old = ∅ :”, are_groups_disjoint(

 'GROUP_YOUNG', 'GROUP_OLD', groups=groups_buggy))

Now identify the misassigned files and remove them from the incorrect group.

import re

def extract_z_values(filenames):

 return [f”Z{m.group(1)}” for f in filenames

  if (m := re.search(r'_Z(∖d+)_', f))]

intersecting = report_group_intersections(

 'GROUP_YOUNG', 'GROUP_OLD', groups=groups_buggy

)

for filename in intersecting:

 groups_buggy['GROUP_Z1'].remove(filename)

 groups_buggy['GROUP_YOUNG'].remove(filename)

print(“Young ∩ Old = ∅ after correction:”,

 are_groups_disjoint('GROUP_YOUNG', 'GROUP_OLD', groups=groups_buggy))

***Note:*** A corrected metadata file is available in the MassIVE archive under updates/2026-03-25_lfnothias_1828a86c/metadata/group_mapping_rosemary_corrected.txt and is used by default in Step 6.

### Problem 3

None of the features of a run appear to have been imported. Related to Step 7.

### Potential solution

Check if the run was accidentally flagged as a blank in the data import. Inspect the sample_types list and verify that it maps correctly to the files list.

### Problem 4

The annotation job completes but no features receive CSI:FingerID structure annotations. Related to Step 12.

### Potential solution

Confirm that the data contains MS/MS spectra. Features without MS/MS data cannot be structurally annotated. Inspect the feature quality distribution: if most included features are LOWEST or BAD, chances are that the MS2 contains an insufficient amount of fragments for structure annotation.

### Problem 5

The fold change job returns an empty table or raises an error. Related to Step 17.

### Potential solution

Verify that both comparison groups contain at least one run each. Use

api.tags().get_groups(project_info.project_id)

to list defined groups and inspect their members.

### Problem 6

The target compound is not found among annotated features. Related to Step 23.

### Potential solution

Inspect all features annotated with the same PubChem CID regardless of adduct type. If the compound is absent entirely, verify that the MS/MS spectrum quality is sufficient and that the feature was not excluded during quality filtering.

### Problem 7

Class-level fold change analysis returns very few classes above the threshold. Related to Step 37.

### Potential solution

Reduce ABS_LOG2_FC_THRESHOLD to inspect the full distribution of class-level fold changes. Also verify that the annotation job completed successfully and that a sufficient number of features received CANOPUS class annotations.

## Resource availability

### Lead contact

Further information and requests for resources should be directed to Markus Fleischauer (markus.fleischauer@uni-jena.de).

### Technical contact

Technical questions on executing this protocol should be directed to Jonas A. Emmert (jonas.emmert@uni-jena.de).

### Materials availability

This protocol does not generate new biological materials.

### Data and code availability

• The rosemary dataset is publicly available on MassIVE: MSV000080553.
• The PySirius client with fold change capabilities is available on GitHub at https://github.com/sirius-ms/sirius-client-openAPI/tree/star-protocol/client-api_python.
• The SIRIUS source code of the version used to replicate the findings shown here is available via https://github.com/sirius-ms/sirius/releases/tag/v6.5.0.
• We provide a Jupyter^20^ notebook that covers everything shown in this protocol on GitHub as well, under https://github.com/sirius-ms/notebooks/blob/main/FoldChange/FoldChangeNotebook.ipynb. Jupyter needs to be installed to run the notebook. Jupyter can be installed in the active conda environment by running pip install jupyter. We use Jupyter version 1.1.1.
• Conda environment files are bundled together with the Jupyter notebook. Including Jupyter, https://github.com/sirius-ms/notebooks/blob/main/FoldChange/environment_jupyter.yaml; excluding Jupyter, https://github.com/sirius-ms/notebooks/blob/main/FoldChange/environment.yaml. Neither file covers the PySirius installation.
• The client source code, Jupyter notebook, and exact SIRIUS build used are archived in Zenodo under https://doi.org/10.5281/zenodo.21257821.

## Acknowledgments

The authors thank Martin A. Hoffmann, Marcus Ludwig, Kai Dührkop, Martin Engler-Lukajewski, Lukas Scholz, and Nils A. Haupt for their help and work around the SIRIUS API and client libraries. The authors have no funding to report.

## Author contributions

J.A.E., M.F., and S.B. conceptualized the study. J.A.E. designed and conducted the experiments. J.A.E. and M.F. developed and implemented the method. J.A.E. wrote the manuscript. S.B. and M.F. provided supervision.

## Declaration of interests

M.F. and S.B. are co-founders of Bright Giant GmbH.

## Declaration of generative AI and AI-assisted technologies in the writing process

During the preparation of this work, the authors used Claude and Codex in order to improve writing as well as code implementations. After using this tool/service, the authors reviewed and edited the content as needed and take full responsibility for the content of the publication.
